# Supplementary material for: Whole-exome sequencing of alpha-fetoprotein producing gastric carcinoma reveals genomic profile and therapeutic targets
Source: Nat Commun. 2021 Jun 24;12:3946. doi: 10.1038/s41467-021-24170-0 (PMC8225795; doi:10.1038/s41467-021-24170-0)
Supplement: Supplementary file 1 — Supplementary Information [file 41467_2021_24170_MOESM1_ESM.pdf]

Whole-exome sequencing of alpha-fetoprotein producing gastric carcinoma reveals genomic profile and therapeutic targets

Supplementary Informations

Supplementary Figures 1- 13

Supplementary Table 1- 8

Supplementary Method

Supplementary References

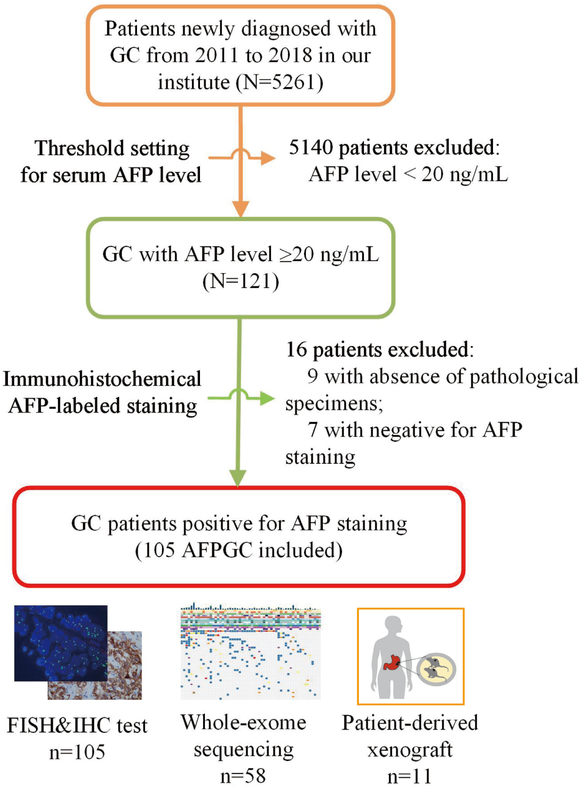

Supplementary Fig. 1. Study workflow. GC, gastric cancer; AFP, alpha-fetoprotein; AFPGC, alpha-fetoprotein producing gastric carcinoma; IHC, immunohistochemistry; FISH, fluorescence in situ hybridization.

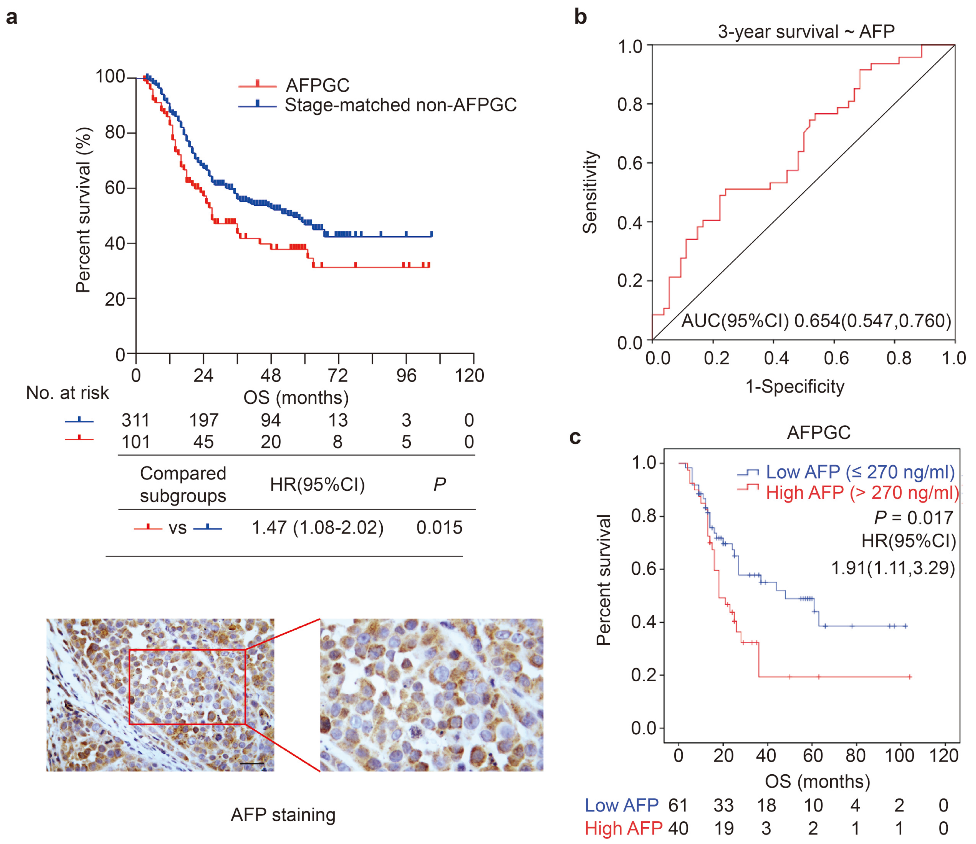

Supplementary Fig. 2. **Survival analysis for AFPGC. (a)** Survival analysis of patients with AFPGC and stage-matched non-AFPGC in our institution. One representative IHC staining of AFP-positive status is shown. **(b)** ROC curve analysis for evaluating the 3-year survival value of serum AFP level. **(c)** The serum AFP level of AFPGC was associated with overall survival. Low AFP level (20 ng/ml ≤ serum AFP ≤ 270ng/ml); High AFP level (270 ng/ml < Serum AFP); Statistical significance was determined using log-rank (Mantel–Cox) test (**a, c**). Non-AFPGC, AFP-negative gastric carcinoma; OS, overall survival; HR, hazard ratio; CI, confidence interval; IHC, immunohistochemistry; AUC, area under the ROC curve; ROC, receiver-operating characteristic.

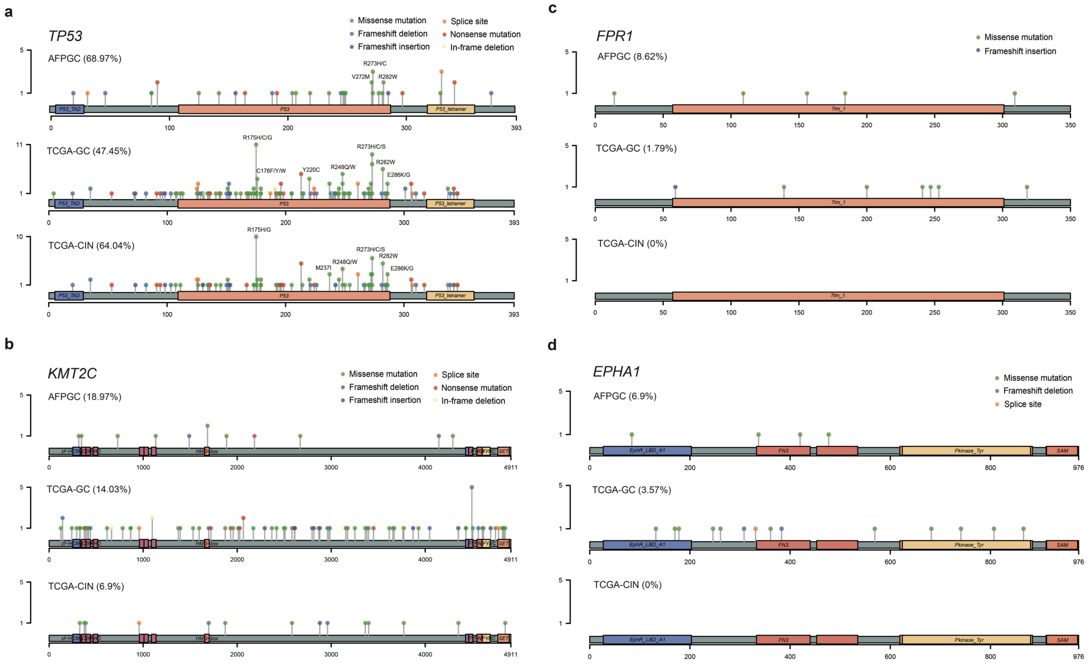

Supplementary Fig. 3. Distribution of non-synonymous somatic mutations in frequently mutated genes in AFPGC, TCGA-GC, and TCGA-CIN. (a) TP53; **(b)** KMT2C; **(c)** FPR1; **(d)** EPHA1**.**

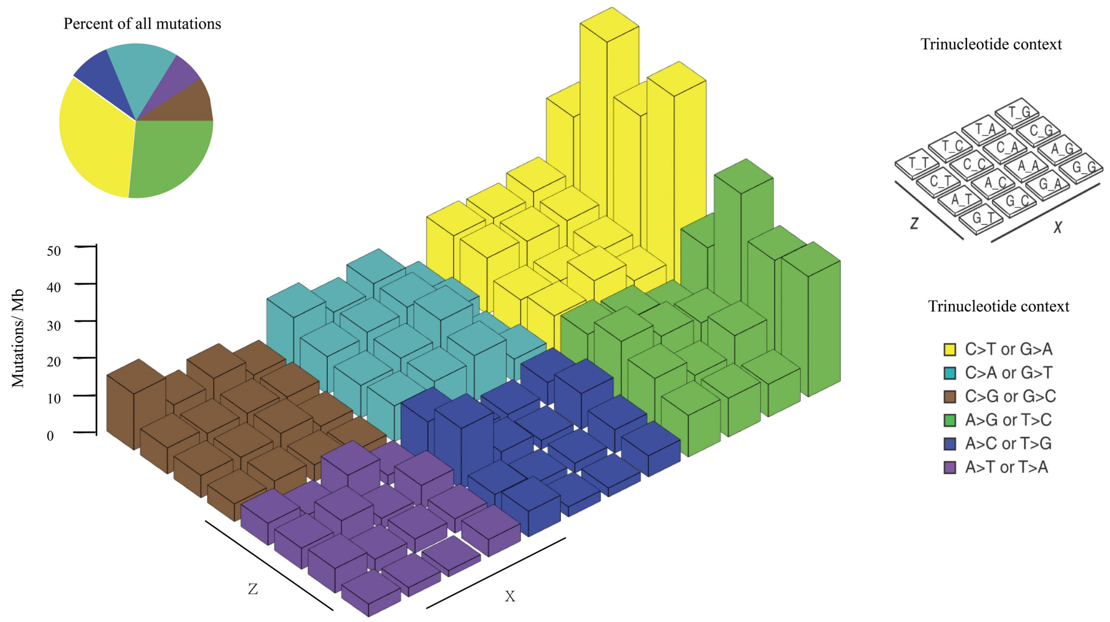

Supplementary Fig. 4. **Mutation spectrum analysis of AFPGC.** Mutation spectrum is derived from 58 paired samples of AFPGC subjected to WES. Base substitutions are divided into 96 mutation types. The height of the bar represents the somatic mutation number of base substitutions.

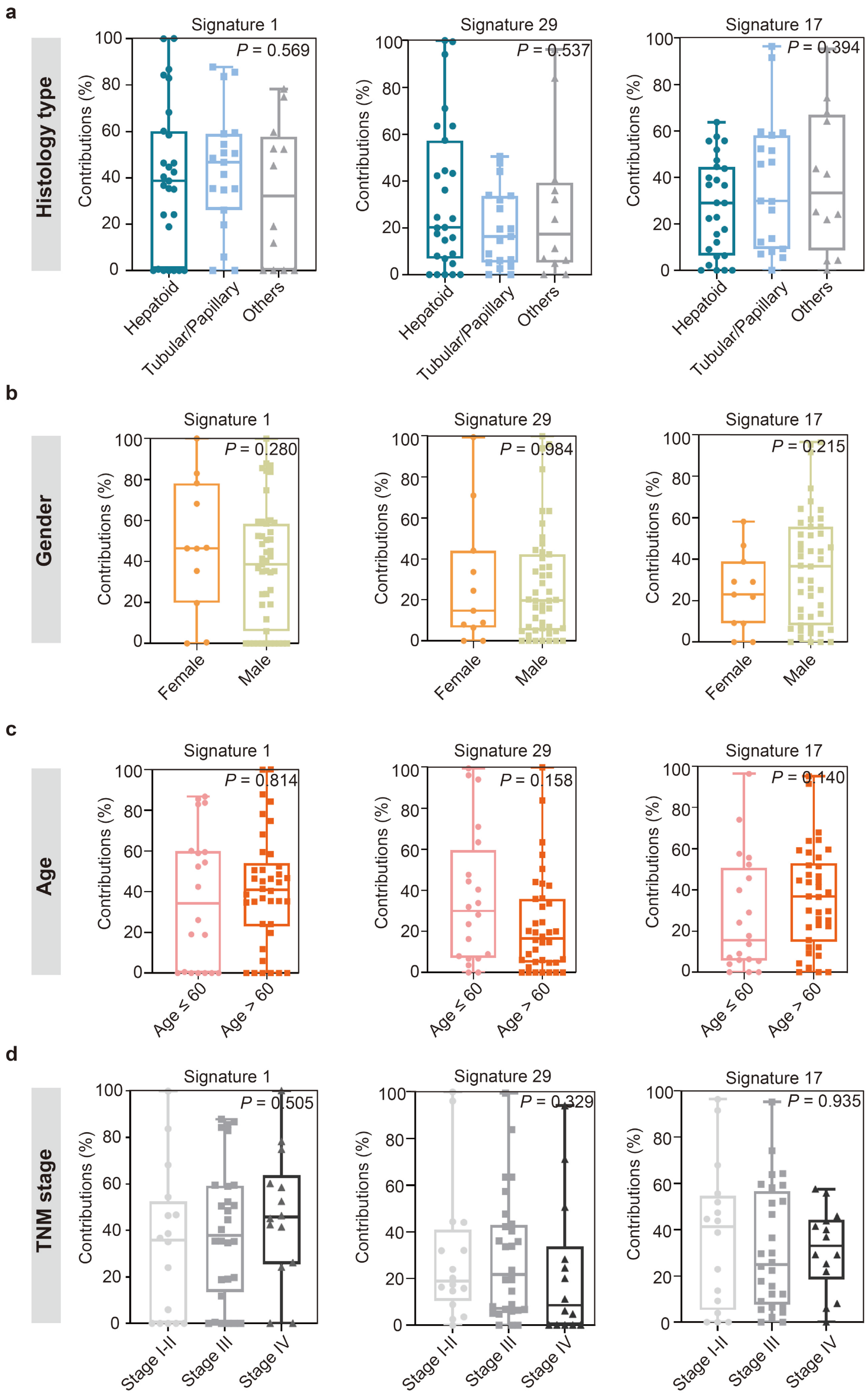

Supplementary Fig. 5. Association between signature contributions and clinicopathological characteristics. (a) Histology type; **(b)** Gender; **(c)** Age; **(d)** TNM stage. Box plots show the median (central line), the 25–75% interquartile range (IQR) (box limits), the ±1.5 times IQR (Tukey whiskers), and all data points, among which the lowest and the highest points indicate minimal and

maximal values, respectively. Statistical significance was determined using Kruskal-Wallis test (a, d) and two-tailed Mann-Whitney U test (b, c). $P < 0.05$ was considered significant.

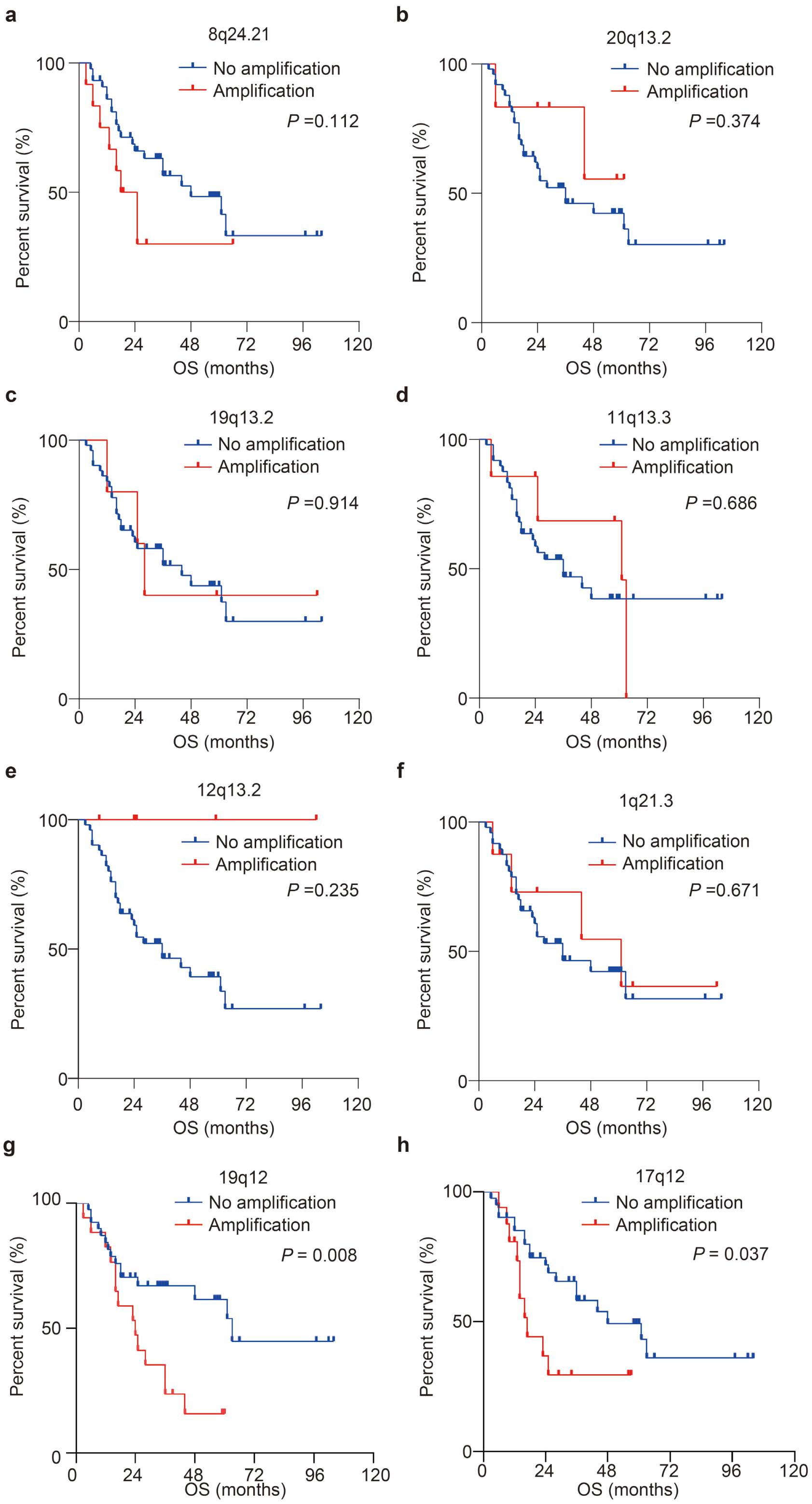

Supplementary Fig. 6. Survival analysis of frequent SCNAs in AFPGC cohort. Patients were divided into two subgroups according to the status of chromosome segment amplification in (**a**) 8q24.21; (**b**) 20q13.2; (**c**) 19q13.2; (**d**) 11q13.3; (**e**) 12q13.2; (**f**) 1q21.3; (**g**) 19q12; (**h**) 17q12. Statistical significance was determined using log-rank (Mantel–Cox) test. OS, overall survival.

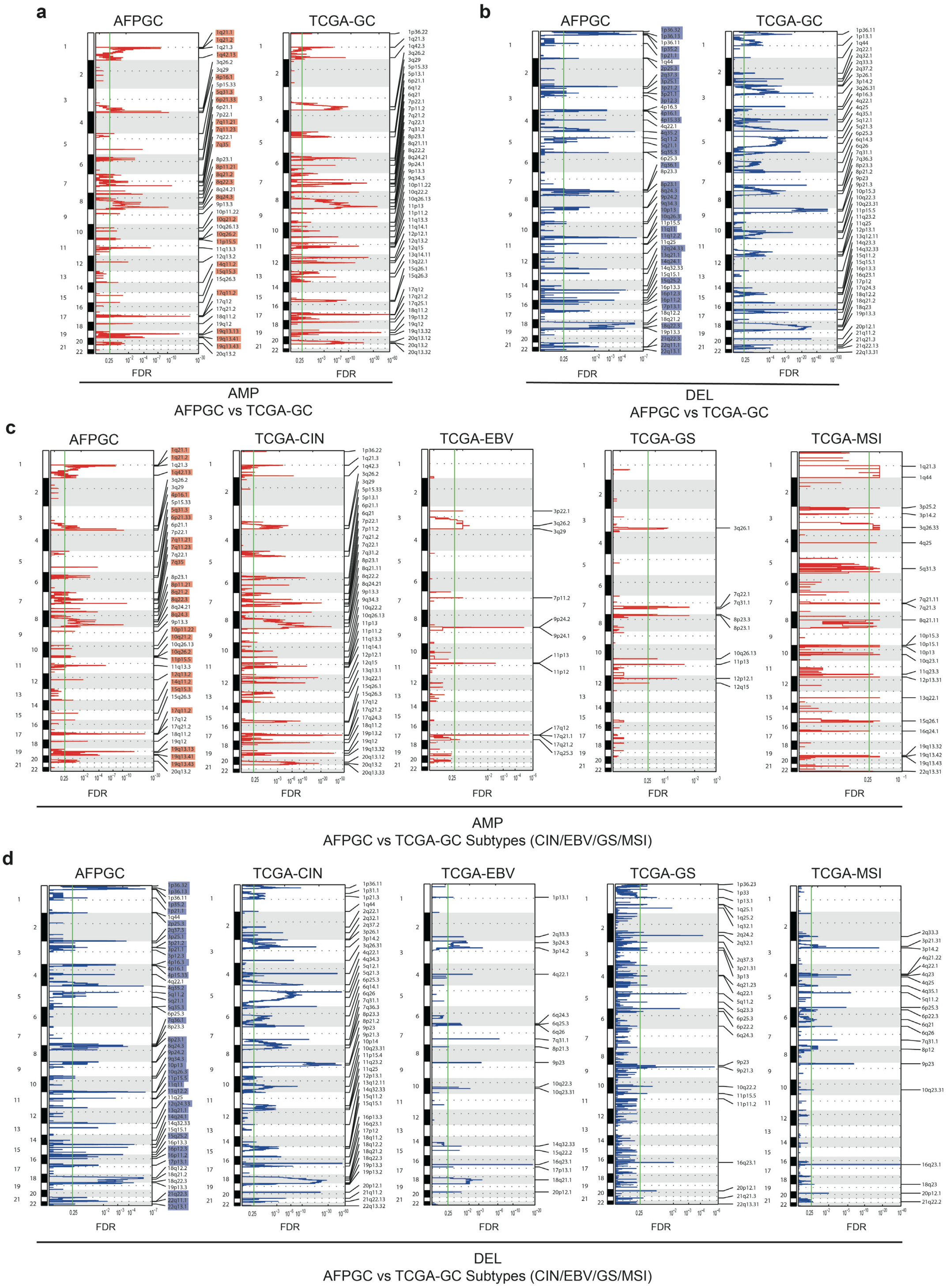

Supplementary Fig. 7. **GISTIC 2.0 significant SCNAs in AFPGC and gastric cancer from TCGA.** The comparison of AFPGC and TCGA-GC in amplifications (**a**) and deletions (**b**). The comparison of AFPGC and TCGA-GC subtypes in amplifications (**c**) and deletions (**d**). Chromosomal locations of peaks of significant focal amplifications (red) and deletions (blue) are plotted by FDR. Annotated regions have an FDR < 0.25, and regions highlighted in red or blue were specific for AFPGC comparing with TCGA-GC or TCGA-CIN. FDR, false discovery rate; AMP, amplification; DEL, deletion; TCGA, The Cancer Genome Atlas; CIN, chromosomal instability; GS, genomically stable; EBV, Epstein-Barr virus; MSI, microsatellite instability;.

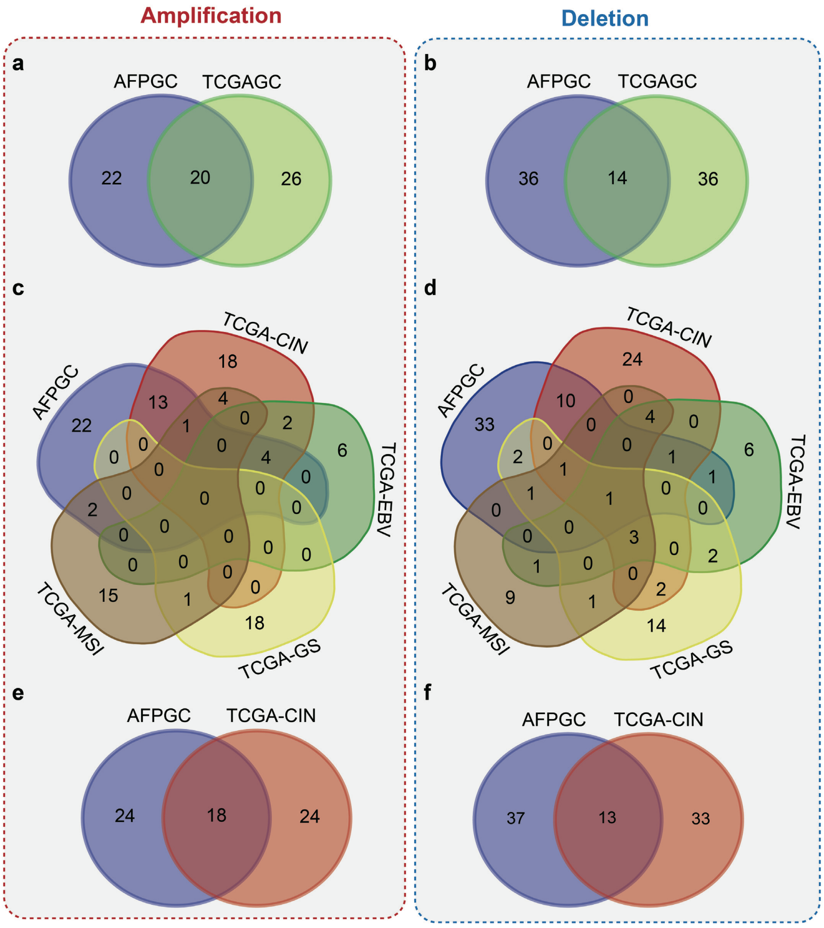

Supplementary Fig. 8. Overlap of significant SCNAs between AFPGC and gastric cancer from TCGA. The Venn diagram displays the joint regions in GISTIC 2.0 amplifications (**a**) and deletions (**b**) between AFPGC and TCGA-GC, amplifications (**c**) and deletions (**d**) between AFPGC and TCGA subtypes, amplifications (**e**) and deletions (**f**) between AFPGC and TCGA-CIN subtype.

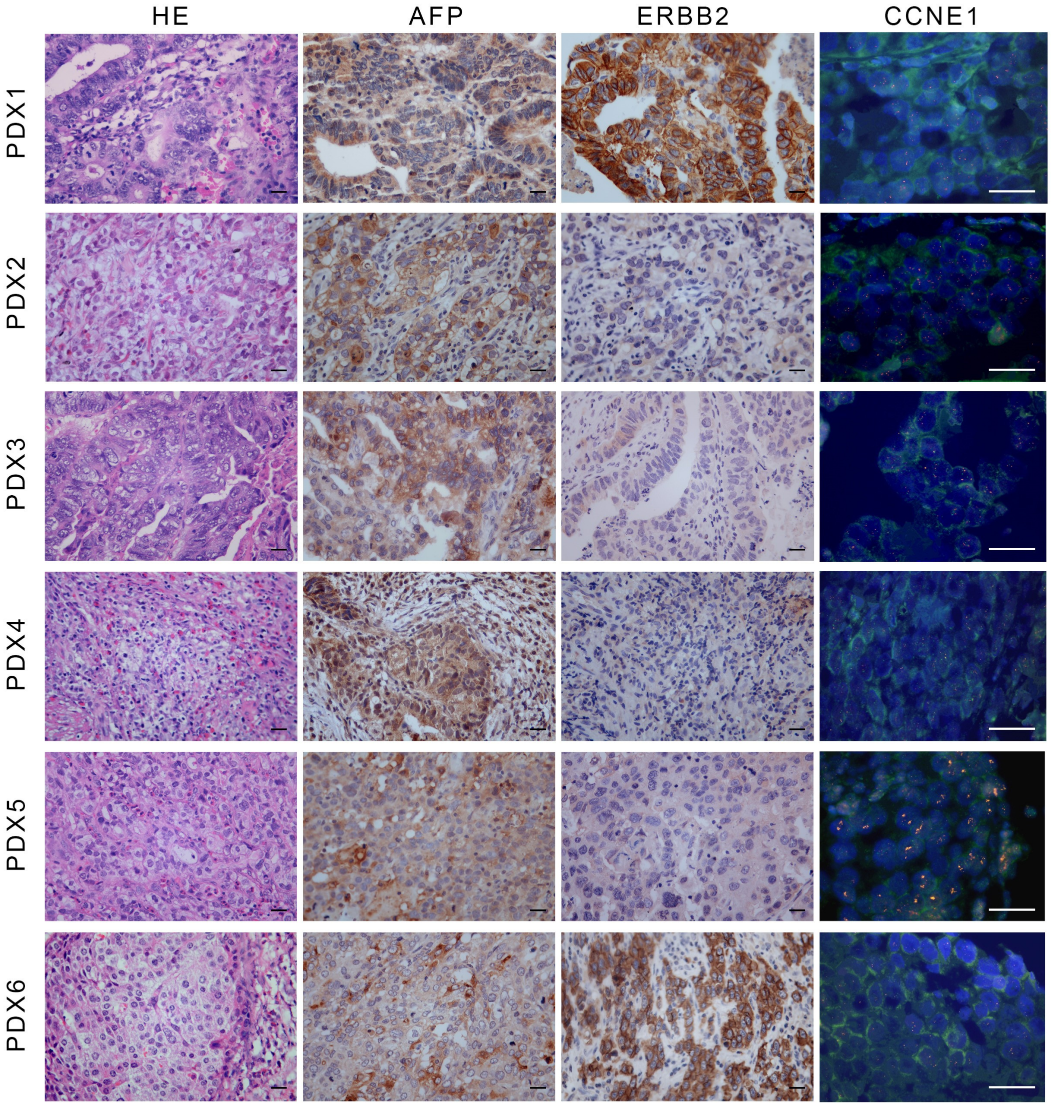
HE
AFP
ERBB2
CCNE1
PDX1
PDX2
PDX3
PDX4
PDX5
PDX6

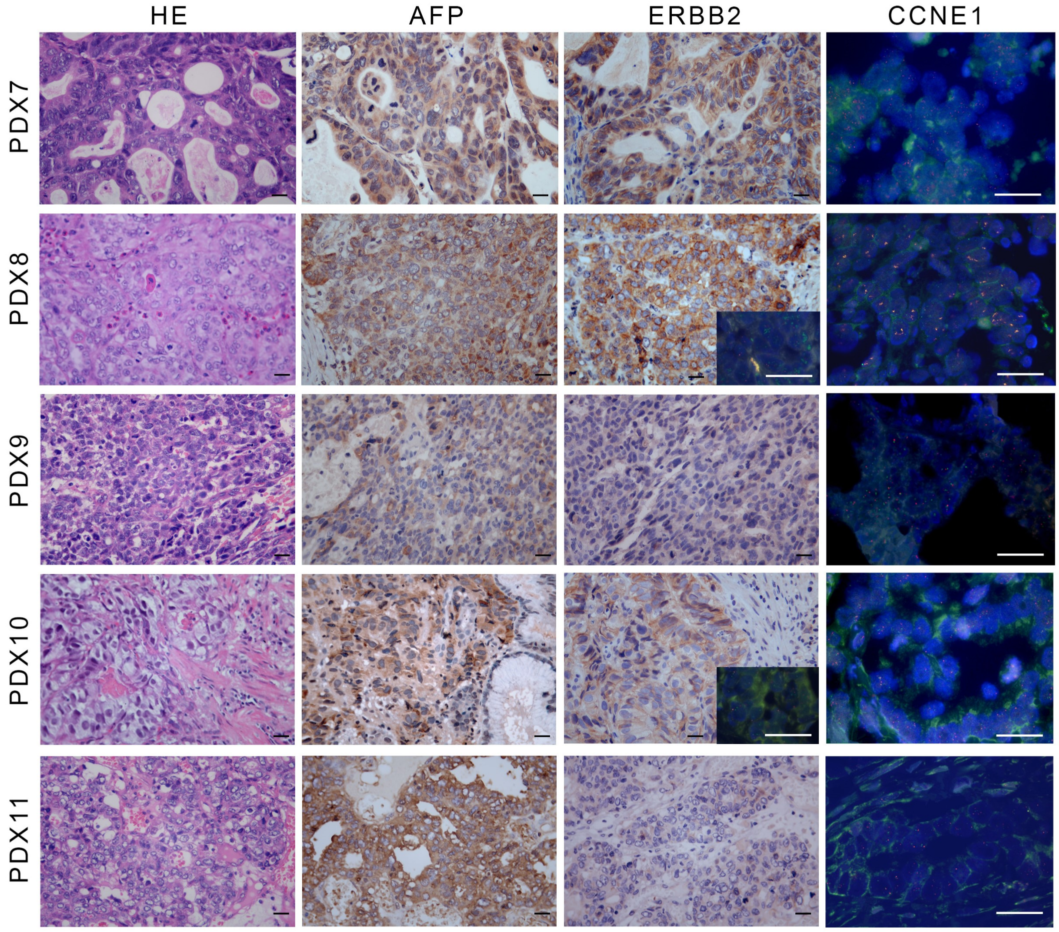

Supplementary Fig. 9. Representatives of pathological and immunohistochemical features of AFPGC PDX models. Presented data are a representative image of three independent experiments. Each row represents a PDX model, and each column represents a pathological or immunohistochemical marker. HE, Hematoxylin and Eosin. Scale bar represents 50 µm.

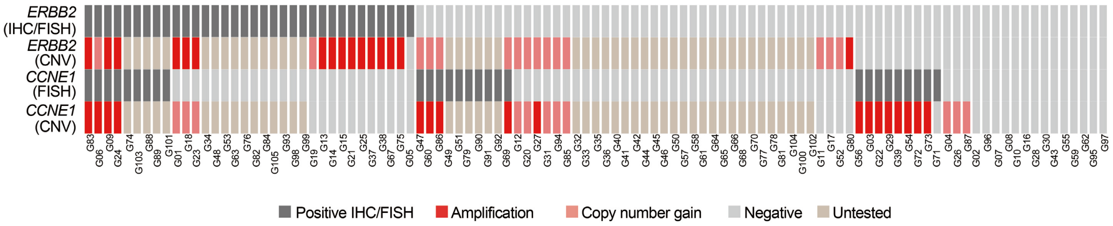

Supplementary Fig. 10. The status of AFP, ERBB2 and CCNE1 in AFPGC cohort. The CNVs status of ERBB2 and CCNE1 are defined by the WES result of 58 patients. IHC, immunohistochemistry; FISH, fluorescence in situ hybridization.

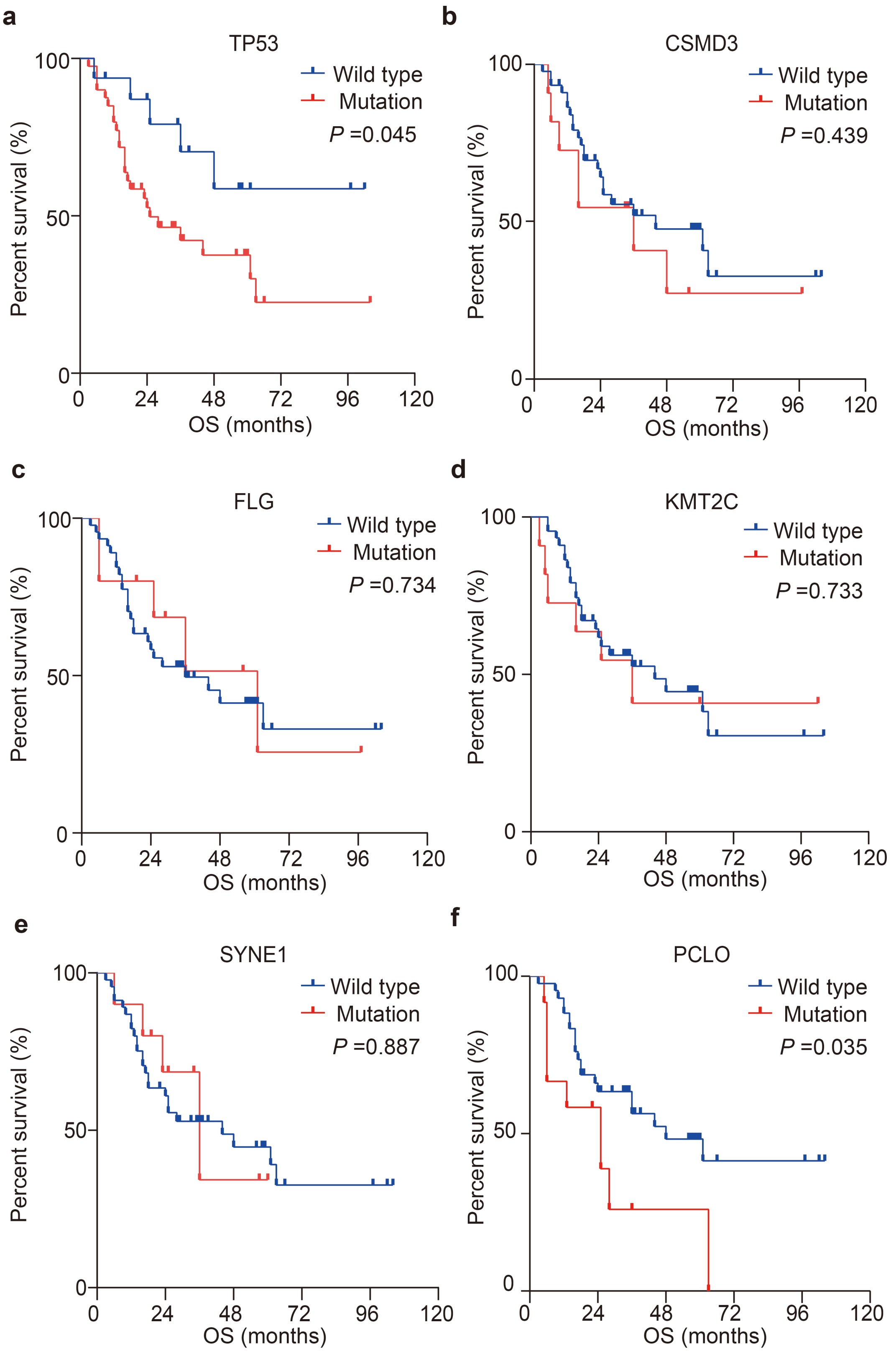

Supplementary Fig. 11. Survival analysis of significantly mutated genes in AFPGC cohort. Patients were divided into two subgroups according to the mutated status of genes in **(a)** TP53**; (b)** CSMD3**; (c)** FLG**; (d)** KMT2C**; (e)** SYNE1**; (f)** PCLO. Statistical significance was determined using log-rank (Mantel–Cox) test. OS, overall survival.

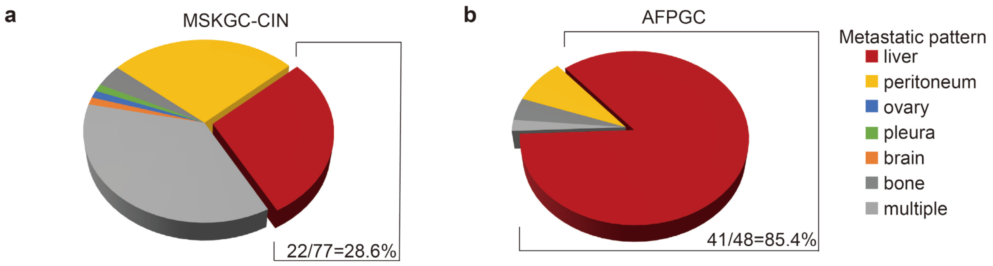

Supplementary Fig. 12. Comparison of metastatic pattern between MSKGC-CIN subtype (a) and AFPGC (b). The data of CIN type in MSKGC cohort was downloaded from cBioportal database; MSKGC, gastric cancer from Memorial Sloan Kettering Cancer Center; CIN, chromosomal instability.

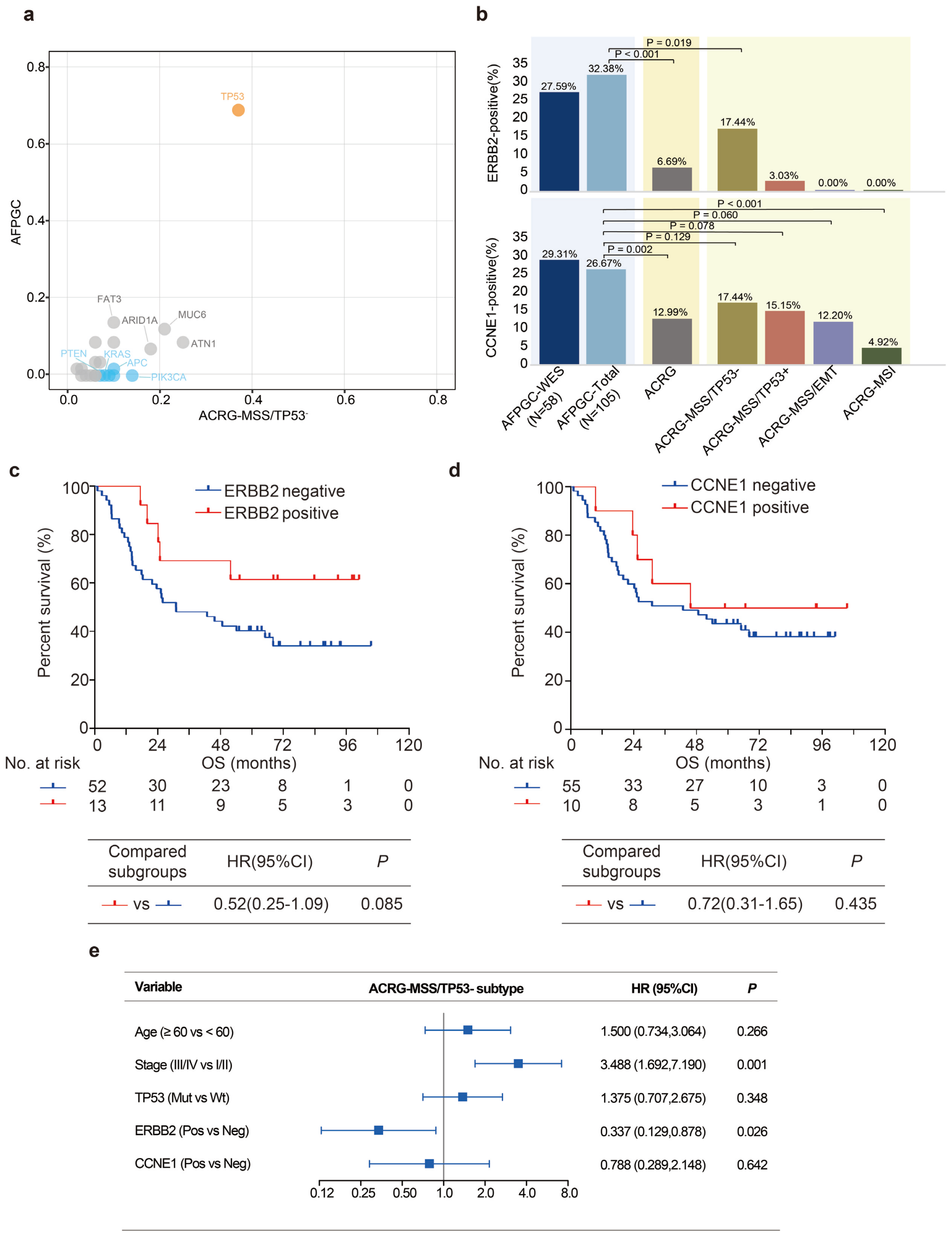

Supplementary Fig. 13. Comparison of mutated genes and copy number alterations in AFPGC and ACRG-MSS/TP53^{-} subtype. (a) The frequency of mutated genes in AFPGC and gastric cancer from ACRG. Orange or blue dots represents the genes in AFPGC with significantly higher or lower mutation frequency than those in ACRG-MSS/TP53^{-} subtype. **(b)** The frequency of ERBB2 and CCNE1 amplifications in AFPGC and gastric cancer from ACRG. **(c-d)** Association between the status of ERBB2 **(c)** or CCNE1 **(d)** and OS in ACRG-MSS/TP53- subtype. **(e)** Forest plot of multivariable cox proportional hazard regression in ACRG-MSS/TP53- subtype. The hazard ratios are presented and the horizontal lines indicate the 95% confidence intervals. Statistical significance was determined using two-sided chi-square test (**b**), log-rank (Mantel–Cox) test (**c, d**), and multivariate COX regression (**e**). ACRG, the Asian Cancer Research Group; MSS, microsatellite stability; MSI, microsatellite instability; EMT, epithelial-mesenchymal transformation; Mut, mutation; WT, wild type; OS, overall survival; HR, hazard ratio; CI, confidence interval; Pos, positive; Neg, negative.

Supplementary Table 1. Clinicopathological characteristics of 105 AFPGC patients.

**Characteristic**	**No. (%) of patients**
**Sex**	
Male	79(75.2)
Female	26(24.8)
**Age (years)**	
Median	64
Range	(30,83)
**Age classification (years)**	
⩾60	78(74.3)
<60	27(25.7)
**Smoking**	
Yes	49(46.7)
No	48(45.7)
Unspecified	8(7.6)
**Operation type**	
Radical	75(71.4)
Palliative	12(11.4)
No surgery	18(17.1)
**Primary lesion site**	
Cardia	24(22.9)
Body	21(20.0)
Antrum	55(52.4)
Unspecified	5(4.8)
**TNM stage**	
I-II	33(31.4)
III-IV	72(68.6)
**Differentiation**	
Hepatoid	46(43.8)
Tubular/Papillary	43(41.0)
Others	16(15.2)
**Lymphovascular invasion**	
Positive	62(59.0)
Negative	29(27.6)
NA*	14(13.3)
**Liver metastasis**	
Yes	42(40.0)
No	62(59.0)
NA**	1(1.0)

NA, not applicable

*The pathologic report did not indicate the presence of lymphovascular invasion

**No follow-up information was available to confirm the presence of liver metastases

Supplementary Table 2. Significantly mutated genes in 58 paired AFPGC samples.

Gene	Indels	SNVs	Total Mutations	Covd Bps	Muts pMbp	P-value LRT	P-value CT	FDR LRT	FDR CT
TP53	7	33	40	108022	370.29	0	0	0	0
PCLO	5	7	12	920762	13.03	0.001787	0.000247	0.13857	0.112819
CSMD3	1	12	13	689107	18.86	3.38E-08	2.03E-09	0.000324	1.95E-05
KMT2C	3	9	12	862080	13.92	0.000121	1.56E-05	0.044304	0.019975
LRP1B	1	10	11	842401	13.06	3.34E-05	6.08E-06	0.023575	0.009938
SYNE1	1	11	12	1591244	7.54	0.003129	0.000288	0.169938	0.125346
FLG	4	9	13	718676	18.09	7.23E-05	1.28E-06	0.036452	0.006118
ZFHX4	1	8	9	638873	14.09	0.012987	0.000398	0.24023	0.155832
COL11A1	0	7	7	354234	19.76	1.75E-06	6.22E-06	0.004638	0.009938
MDC1	3	5	8	378935	21.11	0.00013	4.41E-05	0.046243	0.040205
HCN1	0	5	5	166507	30.03	1.75E-05	0.000175	0.017684	0.089813
PRDM1	1	4	5	179854	27.8	0.002677	0.000512	0.160823	0.171304
ERBB2	1	5	6	250166	23.98	9.81E-05	0.000195	0.041841	0.095898
ERBB4	0	4	4	242316	16.51	0.004741	0.001133	0.19009	0.238644
FPR1	0	5	5	71680	69.75	3.14E-05	3.14E-06	0.023164	0.009938
PRKRIR	1	4	5	130947	38.18	0.000304	3.28E-05	0.063793	0.033108
PCDH18	0	5	5	229370	21.8	0.000268	0.000336	0.060727	0.133974
CHL1	0	5	5	229642	21.77	8.37E-05	0.000123	0.040089	0.07015
SPARCL1	2	2	4	125340	31.91	6.79E-05	0.000264	0.035153	0.117821
SLITRK2	0	4	4	158936	25.17	0.00033	0.000333	0.06535	0.133974
PCDHB2	1	3	4	152261	26.27	0.003683	0.000789	0.177475	0.220696
SLCO4C1	1	3	4	147501	27.12	0.001865	0.001045	0.139631	0.228538
EPHA1	0	4	4	207203	19.3	0.003871	0.001018	0.181289	0.228538
ZNF479	0	3	3	104175	28.8	0.001531	0.000705	0.131213	0.204849
MSR1	2	3	5	111777	44.73	0.000464	5.41E-05	0.078712	0.04319
ATAD2	1	4	5	268948	18.59	0.000665	0.000969	0.092155	0.228538
KRT8	4	0	4	113127	35.36	8.15E-06	0.00056	0.011922	0.174696
OR4K15	0	3	3	60726	49.4	5.62E-05	0.000148	0.031695	0.078746
CHRM2	0	3	3	92839	32.31	0.000162	0.000497	0.050177	0.171304
UGT2B4	0	3	3	125650	23.88	0.000214	0.000896	0.056906	0.228538
CRIPAK	3	2	5	100317	49.84	2.82E-05	2.74E-05	0.021596	0.030915
SNTG1	0	3	3	107743	27.84	0.000367	0.001153	0.06998	0.240087
COL6A1	6	0	6	231990	25.86	2.14E-07	9.34E-05	0.001025	0.057713
RPSA	0	3	3	65878	45.54	6.47E-05	0.000178	0.034463	0.089813

For each gene,Likelihood-Ratio test (LRT) and the Convolution test (CT) were calculated to test the significance of enhanced mutations compared with the background rate and adjusted by false discovery rate (FDR).

Supplementary Table 3. Target therapies of gene alterations and relevant clinical trials in cancer treatment.

Gene	Drug	Clinical trials or preclinical studies (cancer type)	Phase
AKT2	Afuresertib	NCT04374630 (ovarian cancer)	Phase 2
AURKA	BPR1K871	PMID: 27863392 (acute myeloid leukemia)	Preclinical
AXL	Bemcentinib	NCT03824080 (acute myeloid Leukemia)	Phase 2
BCL6	FX1	PMID: 27482887 (lymphoma)	Preclinical
BRCA2	Niraparib	NCT04475939 (non small cell lung cancer)	Phase 3
		NCT04235101 (solid tumor)	Phase 1
CCND1	Abemaciclib	NCT04584853 (breast cancer)	Phase 3
		NCT04238819 (relapsed solid tumor)	Phase 1
CCND3	Abemaciclib	NCT04584853 (breast cancer)	Phase 3
		NCT04238819 (relapsed solid tumor)	Phase 1
CCNE1	Dinaciclib	NCT01580228 (chronic lymphocytic leukemia)	Phase 3
		NCT01434316 (solid tumors)	Phase 1
CDK6	Abemaciclib	NCT04584853 (breast cancer)	Phase 3
		NCT04238819 (relapsed solid tumor)	Phase 1
EGFR	Gefitinib, Erlotinib, Afatinib	Non small cell lung cancer	FDA approved
ERBB2	Trastuzumab	Breast cancer, gastric cancer	FDA approved
ERBB3	Sapitinib	NCT01579578 (metastatic gastric cancer)	Phase 2
ERBB4	Afatinib	Non small cell lung cancer	FDA approved
FGFR2	Futibatinib	NCT04024436 (metastatic breast cancer)	Phase 2
FLT1	Pazopanib	Advanced renal cell carcinoma and soft tissue sarcoma	FDA approved
FLT3	Gilteritinib	NCT02752035/NCT04027309 (acute myeloid leukemia)	Phase 3
IGF1R	Brigatinib	NCT04111705 (metastatic non small cell lung cancer)	Phase 2
MCL1	AZD5991	NCT03218683 (hematologic malignancy)	Phase 1
MYC	MYCi361	PMID: 31679823 (solid tumor)	Preclinical
PAK1	IPA-3	PMID: 32240651 (prostate cancer)	Preclinical
PIK3CA	CH5132799	NCT01222546 (advanced solid tumors)	Phase 1

Supplementary Table 4. The comparison of genomic alterations in potentially targetable genes between AFPGC and TCGA-GC.

	AFPGC (N=58)		TCGA-GC (N=393)		*P*
	Alterations	No alterations	Alterations	No alterations	
	n(%)	n(%)	n(%)	n(%)	
ERBB2	18(31.0)	40(69.0)	73(18.6)	320(81.4)	0.035
CCNE1	17(29.3)	41(70.7)	50(12.7)	343(87.3)	0.002
MYC	13(22.4)	45(77.6)	52(13.2)	341(86.8)	0.072
CCND1	9(15.5)	49(84.5)	29(7.4)	364(92.6)	0.071*
MCL1	8(13.8)	50(86.2)	17(4.3)	376(95.7)	0.009*
FLT1	7(12.1)	51(87.9)	24(6.1)	369(93.9)	0.099*
ERBB3	6(10.3)	52(89.7)	50(12.7)	343(87.3)	0.678
AURKA	6(10.3)	52(89.7)	29(7.4)	364(92.6)	0.430*
AXL	5(8.6)	53(91.4)	27(6.9)	366(93.1)	0.586*
BCL6	5(8.6)	53(91.4)	29(7.4)	364(92.6)	0.789*
BRCA2	5(8.6)	53(91.4)	37(9.4)	356(90.6)	>0.999
EGFR	5(8.6)	53(91.4)	37(9.4)	356(90.6)	>0.999
ERBB4	5(8.6)	53(91.4)	51(13.0)	342(87.0)	0.403
FGFR2	5(8.6)	53(91.4)	30(7.6)	363(92.4)	0.792*
AKT2	4(6.9)	54(93.1)	16(4.1)	377(95.9)	0.308*
PAK1	4(6.9)	54(93.1)	17(4.3)	376(95.7)	0.330*
CCND3	4(6.9)	54(93.1)	26(6.6)	367(93.4)	>0.999*
CDK6	4(6.9)	54(93.1)	36(9.2)	357(90.8)	0.636
FLT3	4(6.9)	54(93.1)	20(5.1)	373(94.9)	0.532*
PIK3CA	4(6.9)	54(93.1)	86(21.9)	307(78.1)	0.007
IGF1R	3(5.2)	55(94.8)	33(8.4)	360(91.6)	0.603*

*P values were from two-sided Fisher’s exact test and the others were from chi-square test, and were significant at < 0.05.

Supplementary Table 5. Comparison of ERBB2 and CCNE1 amplifications in AFPGC and gastric cancer from TCGA.

	AFPGC-105		TCGA-GC			TCGA-CIN			TCGA-EBV			TCGA-GS			TCGA-MSI		
	**AMP**	**No-AMP**	**AMP**	**No-AMP**	**P value#**	**AMP**	**No-AMP**	**P value#**	**AMP**	**No-AMP**	**P value#**	**AMP**	**No-AMP**	**P value#**	**AMP**	**No-AMP**	**P value#**
**ERBB2##**	34	71	46	347	P<0.0001	44	159	P=0.0406	0	63	P<0.0001*	3	24	P=0.0311*	1	43	P<0.0001*
**CCNE1**	28	77	46	347	P=0.0003	37	166	P=0.0661	0	63	P<0.0001*	0	27	P=0.0009*	0	44	P<0.0001*

	AFPGC-58		TCGA-GC			TCGA-CIN			TCGA-EBV			TCGA-GS			TCGA-MSI		
	**AMP**	**No-AMP**	**AMP**	**No-AMP**	**P value#**	**AMP**	**No-AMP**	**P value#**	**AMP**	**No-AMP**	**P value#**	**AMP**	**No-AMP**	**P value#**	**AMP**	**No-AMP**	**P value#**
**ERBB2**	16	42	55	338	P=0.0080	44	159	P=0.3454	0	63	P<0.0001*	3	24	P=0.1028*	1	43	P=0.0004*
**CCNE1**	17	41	46	347	P=0.0001	37	166	P=0.0853	0	63	P<0.0001*	0	27	P=0.0011*	0	44	P<0.0001*

AMP, amplification; No-AMP, No-amplification

*P values were from two-sided Fisher's exact test and the others were from chi-square test, and were significant at < 0.05.

#P values were derived from the comparison between AFPGC and gastric cancer of TCGA (whole cohort or subtypes)

##The status of ERBB2 in AFPGC-105 cohort including ERBB2 FISH positive and ERBB2 amplifications

Supplementary Table 6. Associations of ERBB2 and CCNE1 with clinicopathological parameters of AFPGC (N=105).

Parameters	ERBB2 positive	ERBB2 negative	*P* value	CCNE1 amplification	CCNE1 non-amplification	*P* value
	n(%)	n(%)		n(%)	n(%)	
**Sex**						
Male	23(67.6)	56(78.9)		24(85.7)	55(71.4)	
Female	11(32.4)	15(21.1)	0.212	4(14.3)	22(28.6)	0.134
**Age (years)**						
≥60	25(73.5)	53(74.6)		24(85.7)	54(70.1)	
<60	9(26.5)	18(25.4)	0.902	4(14.3)	23(29.9)	0.106
**TNM stage**						
I-II	6(17.6)	27(38.0)		6(21.4)	27(35.1)	
III-IV	28(82.4)	44(62.0)	**0.035**	22(78.6)	50(64.9)	0.183
**Histological pattern**						
Hepatoid	12(35.3)	34(47.9)		10(35.7)	36(46.8)	
Tubular/Papillary	15(44.1)	28(39.4)		11(39.3)	32(41.6)	
Others	7(20.6)	9(12.7)	0.357	7(25.0)	9(12.7)	0.228
**Lymphovascular invasion**						
Positive	22(84.6)	40(61.5)		19(90.5)	43(61.4)	
Negative	4(15.4)	25(38.5)	**0.033**	2(9.5)	27(38.6)	**0.012**
NA*	8	6		7	7	
**Liver metastasis**						
Yes	19(55.9)	23(32.9)		18(64.3)	24(31.6)	
No	15(44.1)	47(67.1)	**0.025**	10(35.7)	52(68.4)	**0.003**
NA**	0	1		0	1	

NA, not applicable

P values were from two-sided Fisher's exact test and the others were from chi-square test, and were significant at < 0.05.

*The pathologic report did not indicate the presence of lymphovascular invasion

**No follow-up information was available to confirm the presence of liver metastases

Supplementary Table 7. Associations of combined ERBB2 and CCNE1 with clinicopathological parameters of AFPGC (N=105).

Parameters	$ERBB2^{N}CCNE1^{N}$	$ERBB2^{A}CCNE1^{N}$ or $ERBB2^{N}CCNE1^{A}$	$ERBB2^{A}CCNE1^{A}$	*P* value	*P* trend
	n(%)	n(%)	n(%)		
**Sex**				0.983	0.902
Male	39(75.0)	33(75.0)	7(77.8)		
Female	13(25.0)	11(25.0)	2(22.2)		
**Age (years)**				0.499	0.309
⩾60	36(69.2)	35(79.5)	7(77.8)		
<60	16(30.8)	9(20.5)	2(22.2)		
**TNM stage**				**0.047**	**0.015**
I-II	22(42.3)	10(22.7)	1(11.1)		
III-IV	30(57.7)	34(77.3)	8(88.9)		
**Histological pattern**				0.291	**0.044**
Hepatoid	26(50.0)	18(40.9)	2(22.2)		
Tubular/Papillary	21(40.4)	18(40.9)	4(44.4)		
Others	5(9.6)	8(18.2)	3(33.3)		
**Lymphovascular invasion**				**0.004**	**0.001**
Positive	27(54.0)	29(82.9)	6(100)		
Negative	23(46.0)	6(17.1)	0(0)		
NA*	2	9	3		
**Liver metastasis**				**0.001**	**<0.001**
Yes	12(23.5)	23(52.3)	7(77.8)		
No	39(76.5)	21(47.7)	2(22.2)		
NA**	1	0	0		

NA, not applicable

P trend was calculated using linear trend test.

P values were from two-sided Fisher's exact test and the others were from chi-square test, and were significant at < 0.05.

*The pathologic report did not indicate the presence of lymphovascular invasion

**No follow-up information was available to confirm the presence of liver metastases

$ERBB2^{N}CCNE1^{N}$: ERBB2 non-amplification and CCNE1 non-amplification;

$ERBB2^{A}CCNE1^{N}$: ERBB2 amplification and CCNE1 non-amplification;

$ERBB2^{N}CCNE1^{A}$: ERBB2 non-amplification and CCNE1 amplification;

$ERBB2^{A}CCNE1^{A}$: ERBB2 amplification and CCNE1 amplification;

Supplementary Table 8. Clinicopathological characteristics of AFPGC patients corresponding to PDX models

Patient ID	PDX model	Gender	Age	Serum AFP （ng/ml）	TNM Stage (8th)	Differentiation	Lymphovascular invasion	Metastasis condition
G04	PDX9	M	63	121.2	IV	Non-hepatoid	Positive	liver metastasis (Synchronous)
G06	PDX7	M	64	56.6	IV	Non-hepatoid	Positive	liver metastasis (Metachronous)
G09	PDX8	M	58	47.2	IV	Hepatoid	Positive	liver metastasis (Synchronous)
G14	PDX1	M	70	23	IIIB	Non-hepatoid	Positive	No
G22	PDX5	M	62	218.4	IIA	Hepatoid	Positive	No
G23	PDX6	F	62	228	IV	Hepatoid	Positive	liver metastasis (Synchronous)
G26	PDX11	M	69	671	IB	Hepatoid	Negative	No
G39	PDX2	M	73	985.7	IIIA	Hepatoid	Positive	liver metastasis (Metachronous)
G52	PDX3	F	65	1245.8	IIIB	Non-hepatoid	Positive	No
G71	PDX4	M	60	74.5	IIIC	Non-hepatoid	Positive	No
G94	PDX10	M	70	34.5	IB	Non-hepatoid	Unspecified	No

Supplementary Method

In AFPGC-total, Cytoplasmic staining in tumour cells for AFP was evaluated, a positive AFP status was defined as >1% staining of the tumour sectionthe[1]. National Comprehensive Cancer Network (NCCN) guidelines recommend assessment of ERBB2 overexpression using immunohistochemistry (IHC) and ERBB2 amplification using fluorescence in situ hybridization (FISH) or in situ hybridization (ISH). Positive (IHC 3+) or negative (IHC 0 or 1+) of ERBB2 status do not require further FISH testing (Type: evidence based; Quality of evidence: high; Strength of recommendation: strong). When ERBB2 status is equivocal (IHC 2+), FISH testing should be further performed to test the status of ERBB2 amplification. To make a better comparison, FISH testing was further performed on ERBB2 (IHC 3+) samples (22 cases). Previous studies also reported that the correlation between ERBB2 IHC 3+ and ERBB2 amplification in FISH was highly concordant (94%-100% [2,3]). The positive status of CCNE1 was defined as CCNE1 amplification by FISH. The concordance correlation between ERBB2 IHC 3+ and ERBB2 amplification in FISH was highly concordant [2,4].

Supplementary References

1 Murakami, T. *et al.* Clinicopathologic and immunohistochemical characteristics of gastric adenocarcinoma with enteroblastic differentiation: a study of 29 cases. *Gastric Cancer* **19**, 498-507, doi:10.1007/s10120-015-0497-9 (2016).

2 Bang, Y. J. *et al.* Trastuzumab in combination with chemotherapy versus chemotherapy alone for treatment of HER2-positive advanced gastric or gastro-oesophageal junction cancer (ToGA): a phase 3, open-label, randomised controlled trial. *Lancet* **376**, 687-697, doi:10.1016/S0140-6736(10)61121-X (2010).

3 Bartley, A. N. *et al.* HER2 Testing and Clinical Decision Making in Gastroesophageal Adenocarcinoma: Guideline From the College of American Pathologists, American Society for Clinical Pathology, and the American Society of Clinical Oncology. *J Clin Oncol* **35**, 446-464, doi:10.1200/JCO.2016.69.4836 (2017).

4 Bartley, A. N. *et al.* HER2 Testing and Clinical Decision Making in Gastroesophageal Adenocarcinoma: Guideline From the College of American Pathologists, American Society for Clinical Pathology, and the American Society of Clinical Oncology. *Journal of Clinical Oncology* **35**, 446-464, doi:10.1200/jco.2016.69.4836 (2017).
