# Supplementary material for: Whole-exome sequencing of alpha-fetoprotein producing gastric carcinoma reveals genomic profile and therapeutic targets
Source: Nat Commun. 2021 Jun 24;12:3946. doi: 10.1038/s41467-021-24170-0 (PMC8225795; doi:10.1038/s41467-021-24170-0)
Supplement: Supplementary file 3 — Description of Additional Supplementary Files [file 41467_2021_24170_MOESM3_ESM.docx]

**File Name: Supplementary Data 1**

**Description:** The clinicopathological characteristics of AFPGC (n = 105).

**File Name: Supplementary Data 2**

**Description:** Statistics of whole-exome sequencing (WES) performing on the genomic DNA of 58 paired samples in AFPGC.

**File Name: Supplementary Data 3**

**Description:** Somatic SNVs called from 58 paired WES samples.

**File Name: Supplementary Data 4**

**Description:** Comparison of gene mutation frequencies in AFPGC and gastric cancer from TCGA.

**File Name: Supplementary Data 5**

**Description:** Mutational signatures of AFPGC and related previous findings.

**File Name: Supplementary Data 6**

**Description:** Analysis of microsatellite instability status in 58 paired AFPGC samples.

**File Name: Supplementary Data 7**

**Description:** Analysis of somatic copy number alterations (SCNAs) in 58 paired AFPGC samples using GISTIC 2.0.

**File Name: Supplementary Data 8**

**Description:** KEGG pathway enrichment of cancer-driving genes in AFPGC.

**File Name: Supplementary Data 9**

**Description:** Comparison of frequently mutated genes (>10%) between AFPGC and TCGA-GC.

**File Name: Supplementary Data 10**

**Description:** Comparison of frequently mutated genes (>10%) between AFPGC and TCGA-CIN.

**File Name: Supplementary Data 11**

**Description:** TCGA sample list used in this study
