# Supplementary material for: Whole-exome sequencing of alpha-fetoprotein producing gastric carcinoma reveals genomic profile and therapeutic targets
Source: Nat Commun. 2021 Jun 24;12:3946. doi: 10.1038/s41467-021-24170-0 (PMC8225795; doi:10.1038/s41467-021-24170-0)
Supplement: Supplementary file 5 — Reporting Summary [file 41467_2021_24170_MOESM5_ESM.pdf]

nature research

Corresponding author(s): Lisong Teng, Haiyong Wang, Zhan Zhou

Last updated by author(s): Apr 29, 2021

Reporting Summary

Nature Research wishes to improve the reproducibility of the work that we publish. This form provides structure for consistency and transparency in reporting. For further information on Nature Research policies, see our Editorial Policies and the Editorial Policy Checklist.

Statistics

For all statistical analyses, confirm that the following items are present in the figure legend, table legend, main text, or Methods section.

n/a	Confirmed	
☐	☒	The exact sample size (n) for each experimental group/condition, given as a discrete number and unit of measurement
☐	☒	A statement on whether measurements were taken from distinct samples or whether the same sample was measured repeatedly
☐	☒	The statistical test(s) used AND whether they are one- or two-sided<br>*Only common tests should be described solely by name; describe more complex techniques in the Methods section.*
☐	☒	A description of all covariates tested
☐	☒	A description of any assumptions or corrections, such as tests of normality and adjustment for multiple comparisons
☐	☒	A full description of the statistical parameters including central tendency (e.g. means) or other basic estimates (e.g. regression coefficient) AND variation (e.g. standard deviation) or associated estimates of uncertainty (e.g. confidence intervals)
☐	☒	For null hypothesis testing, the test statistic (e.g. F, t, r) with confidence intervals, effect sizes, degrees of freedom and P value noted<br>*Give P values as exact values whenever suitable.*
☒	☐	For Bayesian analysis, information on the choice of priors and Markov chain Monte Carlo settings
☒	☐	For hierarchical and complex designs, identification of the appropriate level for tests and full reporting of outcomes
☒	☐	Estimates of effect sizes (e.g. Cohen's d, Pearson's r), indicating how they were calculated

Our web collection on statistics for biologists contains articles on many of the points above.

Software and code

Policy information about availability of computer code

Data collection	No specific software was used.
Data analysis	The paired-end clean reads were aligned to the Human Genome Reference Consortium build 37(GRCh37)using BWA v.0.7.8. Identification of somatic SNVs was conducted by muTect (1.1.4), and the somatic InDels were detected by Strelka (v1.0.13). ANNOVAR (ANNOVAR_2015Mar22) was used to annotate VCF (variant call format) files. The MuSiC (Genome-Model-Tools-Music-0.04) was used to identify significantly mutated genes from the profiles of somatic SNVs and InDels in AFPGC. According to the frequency of 96 mutation types, the point mutation types were decomposed into several different mutation characteristics by Nonnegative Matrix Factorization(NMF)method (0.22). Then,extracted mutational signatures were compared to the pan-cancer catalog of 30 known signatures referenced in the Catalogue of Somatic Mutations in Cancer (COSMIC) database using Somatic Signatures packages. We identified Somatic copy number alterations (SCNAs) using CNVkit (v6.7) to analyze the copy number state of each tumor. Then we used GISTIC 2.0. Statistical analysis was performed using SPSS 21.0 software.

For manuscripts utilizing custom algorithms or software that are central to the research but not yet described in published literature, software must be made available to editors and reviewers. We strongly encourage code deposition in a community repository (e.g. GitHub). See the Nature Research guidelines for submitting code & software for further information.

Data

Policy information about availability of data

All manuscripts must include a data availability statement. This statement should provide the following information, where applicable:

- Accession codes, unique identifiers, or web links for publicly available datasets
- A list of figures that have associated raw data
- A description of any restrictions on data availability

The raw sequence data reported in this paper have been deposited in the Genome Sequence Archive of Beijing Institute of Genomics, Chinese Academy of Sciences (http://bigd.big.ac.cn/gsa-human/, accession number HRA000429). The whole-exome somatic variants are also publicly available from the European Variation Archive (https://www.ebi.ac.uk/eva, although we have uploaded our data to EVA, we haven't received the confirmation information from EVA.). The clinical data are provided in Supplementary Data 1. A complete list of TCGA cohort can be found in Supplementary Data 8. The somatic mutation data can be found in source data. The MSKCC data are available in the cBioPortal for Cancer Genomics database (https://www.cbioportal.org/study/summary?id=egc_msk_2017). The TCGA data are available in the cBioPortal for Cancer Genomics database (https://www.cbioportal.org/study/summary?id=stad_tcga). TGCA data analyzed for this manuscript were released 28th January 2016. All other data are available within the Article, Supplementary Information or available from the authors upon request. Source data are provided with this paper.

Field-specific reporting

Please select the one below that is the best fit for your research. If you are not sure, read the appropriate sections before making your selection.

☒ Life sciences ☐ Behavioural & social sciences ☐ Ecological, evolutionary & environmental sciences

For a reference copy of the document with all sections, see nature.com/documents/nr-reporting-summary-flat.pdf

Life sciences study design

All studies must disclose on these points even when the disclosure is negative.

Sample size	No sample size was calculated because AFPGC is a rare subtype of gastric cancer. Therefore, we collected all the eligible cases from our institute as many as possible. No power-analyses were done.
Data exclusions	No data exclusions
Replication	Mice were randomly assigned to different groups (5-7 mice/group),Western blot experiment was repeated 3 times with enough reproducibility. Attempts of data replication were successful.
Randomization	Randomization was not applicable to this study, as the patients enrolled in this study were recruited retrospectively, and the clinical data were retrieved from digital medical records.
Blinding	Blinding were not applicable to this study, as the patients enrolled in this study were recruited retrospectively, and the clinical data were retrieved from digital medical records.

Reporting for specific materials, systems and methods

We require information from authors about some types of materials, experimental systems and methods used in many studies. Here, indicate whether each material, system or method listed is relevant to your study. If you are not sure if a list item applies to your research, read the appropriate section before selecting a response.

Materials & experimental systems

n/a	Involved in the study	
☐	☒	Antibodies
☒	☐	Eukaryotic cell lines
☒	☐	Palaeontology and archaeology
☐	☒	Animals and other organisms
☐	☒	Human research participants
☒	☐	Clinical data
☒	☐	Dual use research of concern

Methods

n/a	Involved in the study	
☒	☐	ChIP-seq
☒	☐	Flow cytometry
☒	☐	MRI-based neuroimaging

Antibodies

Antibodies used	Primary antibodies: AFP (ProteinTech , #14550-1-AP), ERBB2 (CellSignal , #2165), AKT (Cell Signal , #4691), p-AKT (Cell Signal , #13038&#xFF09), ERK(Cell Signal , #4695), p-ERK(Cell Signal , #4370), CDK2(ProteinTech , 10122-1-AP), Rb(ProteinTech , 17218-1-AP), p-Rb(Cell Signal , #8516), β-Actin (BOSTER, #BM0627)

	Secondary antibodies: SP Rabbit & Mouse HRP Kit (DAB) (CoWin Biosciences, #CW2069), HRP Conjugated AffiniPure Goat Anti-rabbit IgG (H+L) (BOSTER, #BA1054), HRP Conjugated AffiniPure Goat Anti-mouse IgG (H+L) (BOSTER, #BA1051)
Validation	AFP (ProteinTech , #14550-1-AP) , https://scicrunch.org/resources/Any/search?q=AB_2223933&l=AB_2223933<br>ERBB2 (CellSignal , #2165) ,https://scicrunch.org/resources/Any/search?q=AB_10692490&l=AB_10692490<br>AKT (Cell Signal , #4691) ,https://scicrunch.org/resources/Any/search?q=AB_915783&l=AB_915783<br>p-AKT (Cell Signal , #13038&#xFF09),https://scicrunch.org/resources/Any/search?q=AB_10805010&l=AB_10805010<br>ERK(Cell Signal , #4695) ,https://www.cellsignal.cn/products/primary-antibodies/p44-42-mapk-erk1-2-137f5-rabbit-mab/4695?N=4294956287&Ntt=%234695%29&fromPage=plp<br>p-ERK(Cell Signal , #4370) ,https://www.cellsignal.cn/products/primary-antibodies/phospho-p44-42-mapk-erk1-2-thr202-tyr204-d13-14-4e-xp-rabbit-mab/4370?N=4294956287&Ntt=4370&fromPage=plp<br>CDK2(ProteinTech , 10122-1-AP) ,https://scicrunch.org/resources/Any/search?q=AB_2078556&l=AB_2078556<br>Rb(ProteinTech , 17218-1-AP) ,http://www.ptgcn.com/products/RB1-Antibody-17218-1-AP.htm<br>p-Rb(Cell Signal , #8516) ,https://www.cellsignal.cn/products/primary-antibodies/phospho-rb-ser807-811-d20b12-xp-rabbitmab/8516?N=4294956287&Ntt=8516&fromPage=plp<br>β-Actin (BOSTER, #BM0627), http://www.boster.com.cn/product/anti-actin-antibody_bm0627.html<br>SP Rabbit & Mouse HRP Kit (DAB) (CoWin Biosciences, #CW2069), https://www.cwbiotech.com/goods/index?id=10297<br>HRP Conjugated AffiniPure Goat Anti-rabbit IgG (H+L) (BOSTER, #BA1054), http://www.boster.com.cn/product/hrp-conjugated-affinipure-goat-anti-rabbit-igg-h-l_ba1054.html<br>HRP Conjugated AffiniPure Goat Anti-mouse IgG (H+L) (BOSTER, #BA1051), http://www.boster.com.cn/product/hrp-conjugated-affinipure-goat-anti-mouse-igg-h-l_ba1051.html

Animals and other organisms

Policy information about studies involving animals; ARRIVE guidelines recommended for reporting animal research

Laboratory animals	Four-to-six-week-old female BALB/c nude mice, purchased from Shanghai Slac Laboratory Animal Corporation (Shanghai, China), were housed with regular 12-hour light/12-hour dark cycles for at least three days before use. Ambient temperature was 20 ~ 22°C, kept at constant humidity of 40 ~ 60%.
Wild animals	No wild animals were used in the study.
Field-collected samples	No field collected samples were used in the study.
Ethics oversight	Animal care and experiments were performed under the approval and supervision of the Animal Experimental Ethical Inspection of the First Affiliated Hospital, College of Medicine, Zhejiang University (No.2018-378).

Note that full information on the approval of the study protocol must also be provided in the manuscript.

Human research participants

Policy information about studies involving human research participants

Population characteristics	From January 2011 to December 2018, there were 121 cases of primary gastric carcinoma with elevated serum AFP levels (≥20 ng/ml) at diagnosis at the First Affiliated Hospital, School of Medicine, Zhejiang University. 105 patients with AFP-positive immunohistochemical staining were finally enrolled. For comparison, we randomly selected 1:3 cases (311 patients) with stage-matched primary gastric cancers with normal serum AFP levels in our institution. Among the 105 AFPGC patients, 79 (75.2%) were males, with the median age at diagnosis was 64 years (range: 30-83 years)
Recruitment	105 patients with AFP-positive immunohistochemical staining at the First Affiliated Hospital, School of Medicine, Zhejiang University were finally enrolled. We retrospectively collected fresh-frozen or formalin-fixed paraffin-embedded (FFPE) tumor tissues and matched tumor adjacent normal tissues from 58 AFPGC patients for genomic characterization, after excluding 47 patients with prior chemotherapy (23 patients), insufficient tumor volume (12 patients), and low quality of DNA (12 patients). Because of the rarity of AFPGC, we enrolled as many patients as possible in this study. No selection-bias was present.
Ethics oversight	Patients derived paraffin-embedded tissue samples were used in accordance with ethical guidelines in the First Affiliated Hospital, School of Medicine, Zhejiang University (No.2018-309).

Note that full information on the approval of the study protocol must also be provided in the manuscript.
