# Supplementary figures and images for: Whole-exome sequencing of alpha-fetoprotein producing gastric carcinoma reveals genomic profile and therapeutic targets
Source: Nat Commun. 2021 Jun 24;12:3946. doi: 10.1038/s41467-021-24170-0 (PMC8225795; doi:10.1038/s41467-021-24170-0)

ERBB2

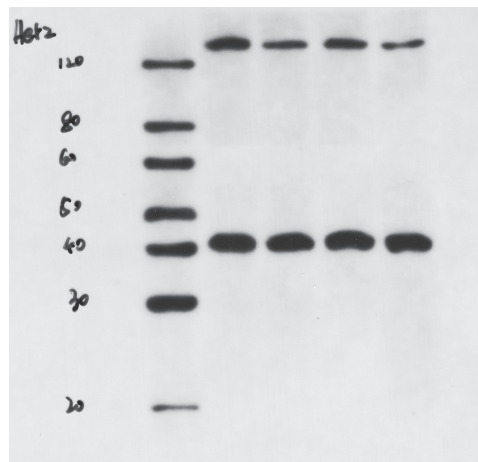

ERK

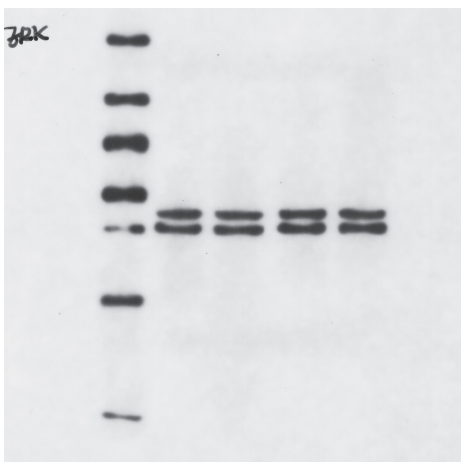

p-ERK

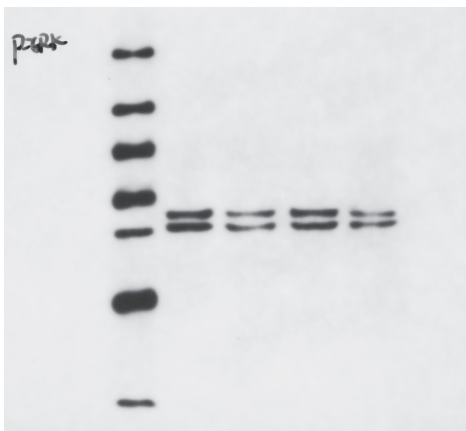

AKT

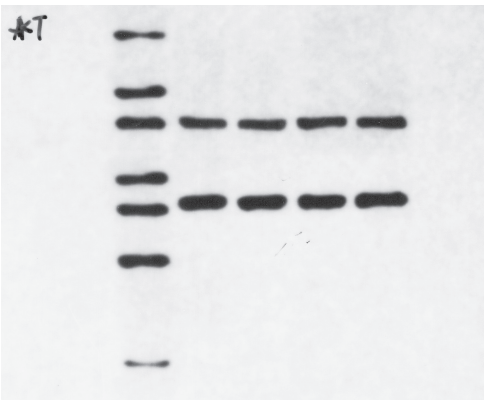

p-AKT

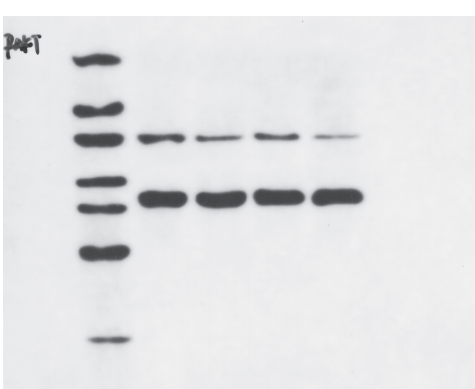

CDK2

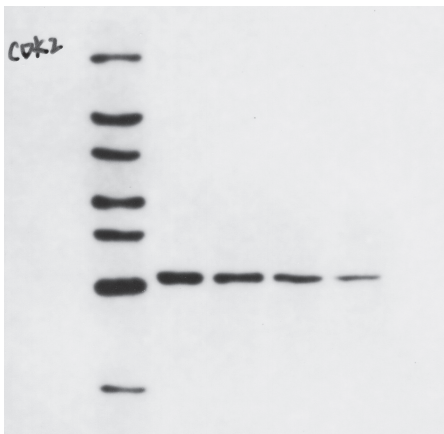

Rb

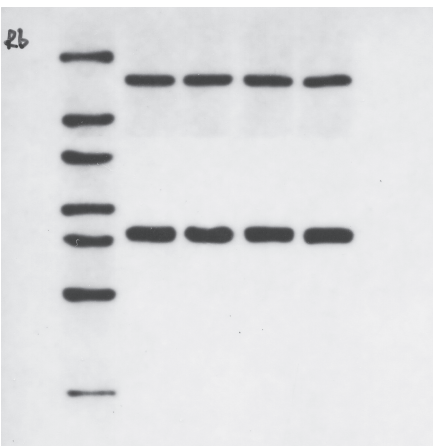

p-Rb

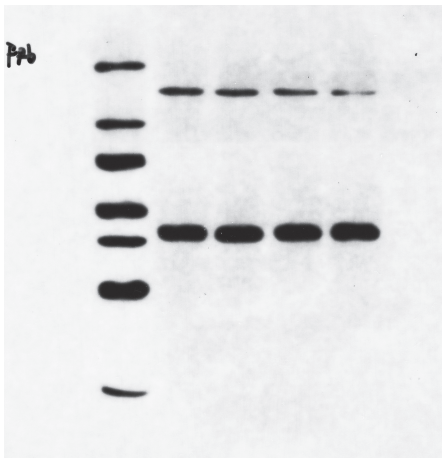

$\beta$ -actin

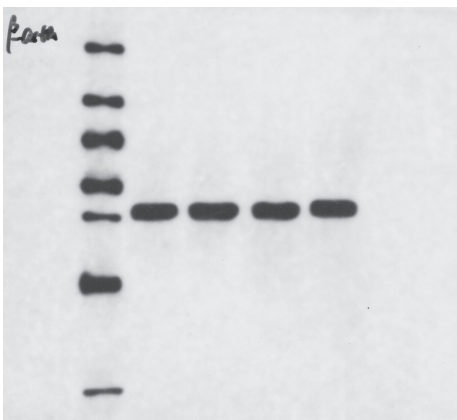

Supplement: Supplementary file 6 — Source Data [file 41467_2021_24170_MOESM6_ESM.zip › Source data2-full scan of blot in figure 5l.pdf]
